# Psychotropic medication for challenging behavior in people with learning disabilities in Qatar

**DOI:** 10.1192/j.eurpsy.2021.1910

**Published:** 2021-08-13

**Authors:** Y. Eltorki, M. El Tahir, B. El Husein, O. Abdallah

**Affiliations:** Psychiatry, Hamad Medical Corporation, Doha, Qatar

**Keywords:** Learning disabilities

## Abstract

**Introduction:**

Challenging behavior is a common reason for referral to psychiatric service. Psychotropic medications widely used to modify behaviors, even when no evidence of diagnosable mental illness. However, literature show little evidence that benefits outweigh the risks in their prescription. Monitoring using International guidelines may help improving the outcomes. We audit current practice against known standards.

**Objectives:**

- To assess adherence within the Qatar Mental Health Services to National guidelines on using medication to manage behavior problems in adults with a learning disability. - To identify strengths and weaknesses in current practice. - To Make recommendations to improve LD patient care

**Methods:**

Patients with LD attending psychiatric clinic screened using selection and exclusion criteria and data collected and analyzed using format from the International standards.

**Results:**

102 patients screened, 85 selected and 17 cases excluded Age range 18 to 50 years. 27% mild, 29% moderate and 44% severe LD, Autism 40% Psychiatric Diagnosis 55% Challenging behavior 45% Antipsychotic prescribing: 79% Rationale documented in 74%, Capacity assessment in 81%, Review of side effects in 53% Safety of medication in 61%, Medication discontinuation in 66%, Reasons for discontinuation in 36%

**Conclusions:**

Antipsychotics use (79%) is high with several combinations of IM and oral or more than 2 drugs. There is need for improvement across all standards. Rationalising the prescribing for LD patients to improve the outcomes for the safety of these patients. The audit indicate need for specialist service to monitor prescribing and apply standards of care in clinical service.

**Disclosure:**

No significant relationships.

